# Peer pressure induced punishment resolves social dilemma on interdependent networks

**DOI:** 10.1038/s41598-021-95303-0

**Published:** 2021-08-04

**Authors:** Kaipeng Hu, Yewei Tao, Yongjuan Ma, Lei Shi

**Affiliations:** 1 School of Statistics and Mathematics, Yunnan University of Finance and Economics, Kunming, 650221 China; 2 School of Statistics and Mathematics, Shanghai Lixin University of Accounting and Finance, Shanghai, 201209 China

**Keywords:** Complex networks, Nonlinear phenomena, Statistical physics

## Abstract

Despite the fruitful evidence to support the emergence of cooperation, irrational decisions are still an essential part of promoting cooperation. Among the many factors that affect human rational decision-making, peer pressure is unique to social organisms and directly affects individual cooperative behaviors in the process of social interaction. This kind of pressure psychologically forces individuals to behave consistently with their partners, and partners with inconsistent behaviors may suffer psychological blows. As feedback, this psychological harm may in turn affect individual cooperative decisions. There is evidence that when peer pressure exists, partnerships can reduce free-riding in enterprise. Based on interdependent networks, this paper studies the impact of peer pressure on cooperation dynamics when the strategies of corresponding partners from different layers of the networks are inconsistent. We assume that when individuals are under peer pressure, their payoffs will be compromised. The simulation results show that the punishment effect will force the expulsion of partners with different strategies, which will further reduce the proportion of partners with inconsistent strategies in the system. However, in most cases, only moderate fines are most conductive to the evolution of cooperation, and the punishment mechanisms can effectively promote the interdependent network reciprocity. The results on the small world and random network prove the robustness of the result. In addition, under this mechanism, the greater the payoff dependence between partners, the better the effect of interdependent network reciprocity.

## Introduction

Nowadays, with the tremendous increase in social productivity, the basic material requirements of some people have been basically met. At the same time, more and more people are beginning to pay attention to spiritual issues. In particular, sociologists realize that mental stress may also affect the further development of society. Peer pressure (or social pressure) is an influence unique to social animals and the most common social psychological state^1–5^. It implies that individuals will be influenced by society and others when making choices. Rigorous, peer pressure is the direct influence of peers on individuals, and encourages peers or individuals to change their attitudes, values, or behaviors to make them consistent with influential groups or individuals. Due to the prevalence of peer pressure in social groups, the improvement of social interests and the guidance of correct values are inseparable from the rational use of peer pressure.

In social dilemmas, an individual’s rational strategy is defection to maximize personal interests, while the maximization of collective interests requires individuals’ cooperation^6–8^. Thus, it is a challenge to resolve social dilemmas among rational individuals. Different from basic assumptions, the driving force of personal behavior and decision-making is very complex, among which irrationality is one of the important factors^9^. For example, the cognitive bias promotes cooperation in social dilemma experiments^11^. It has been proved that the existence of peer pressure may have impacts upon the rational judgment of individuals and can further affect the emergence of cooperation^10^. In addition, behavioral convergence is an important essence of peer pressure to affect the evolution of cooperation^12,13^. Group psychology is a typical product of the convergence of social behavior caused by peer pressure^14,15^, conformists may even abandon their personal goals, and choose to be consistent with the group^16–19^. In previous research^20–23^, scholars revealed that the presence of conformists may be beneficial for the resolution of social dilemmas. However, the reason for choosing the most common strategy is to ensure that the individual’s return will not be much lower than average^20^. Therefore, the conformist mental is essentially an income-related convergence effect, and the punishment in payoffs is a direct effect of peer pressure, and may also lead to the convergence of social behavior. It is worth mentioning that, the conformity chases the vast majority of the group, and the psychological punishment caused by social pressure may be more affected by a small number of individuals with close relationships.

In addition, the network structure also plays an important role in individual decision-making^25–30^. Especially, evolutionary games on multi-layer networks have attracted much attention^31–36^. Unlike on isolated networks, the interactions on the multilayer networks have additional coupling effects on decision-making. For example, Su et al. provided an understanding of three-layer networks reciprocity in evolutionary public goods game, one layer for investment, another layer for allocation and the remaining layers for imitation^37^. Wang et al. found that breaking the symmetry through assortative mixing in one layer or both layers, impedes the evolution of cooperation^38^. Among the three classical multi-layer networks models^39^, the interdependent networks have gained more attention in the field of evolutionary game dynamics. In evolutionary spatial games, a utility function is usually used to couple the individuals’ payoffs on each layer of networks to further establish interdependence relationships. For example, Maja et al. proposed a new path to study the problem of associativity between two-layer networks, they find that under certain conditions the coupling relationship between two layers of the networks can facilitate cooperative behavior^40^.

From a realistic perspective, when an individual’s views or behaviors are inconsistent with his partners, they are usually susceptible to peer pressure and can further lead to negative emotions. In our present work, we abstract the negative emotions as punishments on individuals’ payoffs. Specifically, if one’s strategy is different from their partner in another layer of the network, then the payoffs of both will be reduced to ($1-u$) times the original ($0 \le u \le 1$). In “Methods” section, we describe the punishment mechanism induced by peer pressure. “Results” introduces our observations; in “Discussion”, we summarize this work and discuss its implications.

## Results

**Figure 1:**
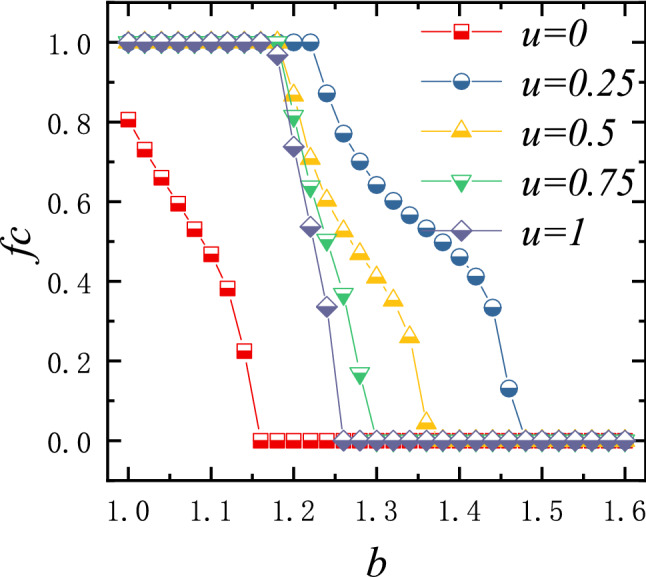
The stationary fraction of cooperators as a function of *b* for $u = 0$, 0.25, 0.5, 0.75 and 1. Results obtained on interdependent lattice with $L = 200$ and $\alpha = 0.5$.

We start by exploring the impact of the punishment factor *u* on the evolution of cooperation. Figure 1 shows cooperation rate as a function of the temptation to defect *b* for different values of *u*. Since the punishment imposed on the individuals on the two-layers (in the following content, we call it layer *A* and layer *B*) networks are symmetric, the cooperation rates of the individuals on the two-layer networks are also consistent. For simplicity, the quantitative results in this paper are the average of the data obtained on the two-layer networks.

**Figure 2:**
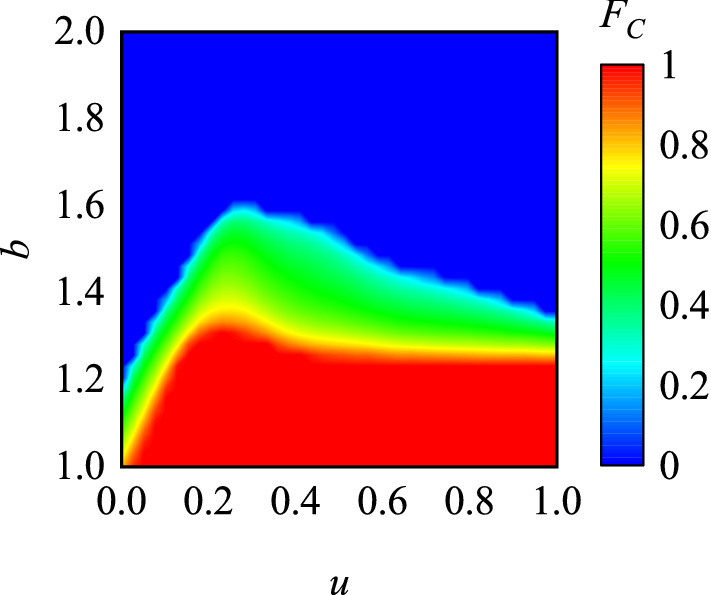
The color map encodes the stationary fraction of cooperators dependent on the temptation to defect *T* and the punishment factor *u*. Results obtained with $L = 200$ and $\alpha = 0.5$.

From Fig. 1, we can see that cooperation frequency monotonously declining with increasing *b* for all the values of *u*. When $u = 0$, the punishment mechanisms are invalid, and cooperation can be maintained until $b = 1.2$. Obviously, the maintenance of cooperation mainly depends on the reciprocal relationship on the interdependent networks. However, when peer pressure is introduced ($u \ge 0$), cooperation can be effectively promoted. Especially, when $u = 0.25$, the cooperation rate can be maintained to $b = 1.48$, and when $b \le 1.2$, cooperation can completely dominate the system. Therefore, punishment caused by peer pressure is conductive to the evolution of cooperation, but the level of cooperation shows a non-monotonous dependence on the fines. From the perspective of punishment, the influence of the mechanism in this paper on cooperation is consistent with the previous conclusions^41^.

**Figure 3:**
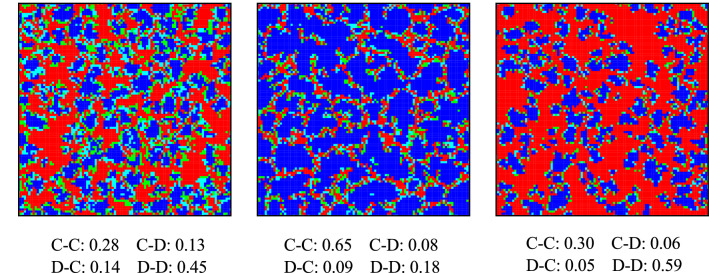
Stable spatial distribution of strategies couple for $u = 0.1$ (left panel), 0.2 (middle panel), 0.6 (right panel). C–C, C–D, D–C and D–D couples are colored in blue, green, light blue and red, where a C–D couple means that the player on layer *A* chooses cooperation and his partner on layer *B* chooses defection, and other notations can be defined similarly. It can be seen both from the snapshots and additional data on the ratio of strategy couples that the proportion of interdependent individuals with inconsistent strategies decreases as u increases. Results obtained with $b = 1.3$, $L = 100$, and $\alpha = 0.5$.

The results in Fig. 1 cannot fully explain how the values of *u* affects the evolution of cooperation. Therefore, we show the average cooperation rate as a function of *u* and *b* in Fig. 2. It is very clear that, for $b < 1.25$, as *u* increases, the cooperation rate monotonically increases until it reaches a state of full cooperation. For $1.25 \le b \le 1.6$, there is an optimal value region of *u* (around $u = 0.2$) in which cooperation can be most effectively promoted. Specifically, as *u* increases, the cooperation rate goes through a process of first increasing and then decreasing, and reaches the highest cooperation level near $u = 0.2$. When the values of *b* fall in other ranges, the large dilemma strength^42^ will completely hinder the spread of cooperation regardless of the value of *u*. From Fig. 2, we can summarize that the setup of punishment is conductive for cooperation, but excessive fines are not the best option.

**Figure 4:**
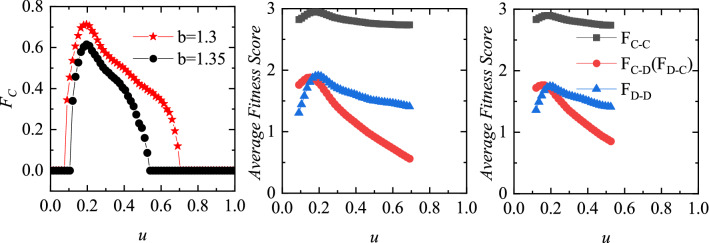
Left panel features the fraction of cooperators as a function of the punishment factor *u*. Middle and right panel features the averaged fitness of competing couples (the individuals whose neighbors hold different strategies) as a function of *u* for $b = 1.3$ and $b = 1.35$, respectively. It is worth mentioning that when the system reaches full cooperation or full defection, there is no such individuals in the system, and no data will be displayed in the middle and right panel. Results obtained with $L = 200$ and $\alpha = 0.5$.

**Figure 5:**
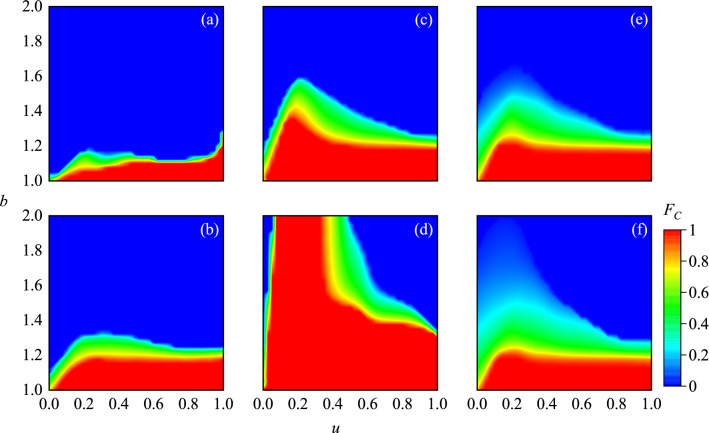
The color map encodes the stationary fraction of cooperators in dependence on the temptation to defect *T* and the punishment factor *u*. panel (**a**–**d**) are obtained with parameter $\alpha =0,0.3,0.6,0.9$, respectively. Panel (**e**,**f**) are obtained on regular small world networks and regular random networks for $\alpha =0.5$. All the results obtained with $L = 200$.

We monitor the typical snapshots to observe the impact of punishment effect from a micro perspective in Fig. 3. It shows the distribution of strategies on the two-layer networks in stable state for $u = 0$(left panel), 0.2(middle panel), and 0.6(right panel). Due to the same structure of the two-layer networks, four colors are sufficient to show all possible strategy distributions. Specifically, C–C, C–D, D–C and D–D couples are colored in blue, green, light blue and red, where C–D couple means that the player on layer *A* chooses cooperation and his partner on layer *B* chooses defection, and other notations can be defined similarly. We find that the slight quantitative difference in the cooperation rate shown in the above results correspond to the considerable difference in the distribution of strategies. In the left panel of Fig. 3, C–C couples and D–D couples are separated by a large number of C–D couples and D–C couples. In the middle panel, C–D couples and D–C couples are much less, only resulting in a narrow barrier between the C–C clusters and the D–D couples. In the right panel, the obstacles used to isolate the C–C couples and the D–D couples are further reduced, and a large number of C–C couples are directly exposed around the D–D couples. From left to right panel, the peer pressure effects become larger, and the partners who hold different strategies will suffer a larger fine. Therefore, the proportion of C–D couples and D–C couples are decreasing from left to right panel (this can also be observed in the additional data under the corresponding snapshot). Comprehensively considering the result above, C–D and D–C couples play a decisive role in the spreading of strategies and the evolution of cooperation. Specifically, C–D and D–C couples can protect C–C couples from being invaded by D–D couples, however, at the same time, their existence also hinders the expansion of C–C couples. Therefore, cooperation can be effectively promoted only when the fraction of C–D couples to D–C couples is moderate.

As individuals’ fitness score is a direct driver of strategy transition, we next explain the evolution of cooperation from the perspective of the average fitness. The left panel of Fig. 4 presents the fraction of cooperators on the two layers of lattice as a function of *u*. Obviously, a moderate value of *u* ($u = 0.2$) maximizes cooperation rate both for $b = 1.3$ and $b = 1.35$, which is consistent with the aforementioned phenomenon in Fig. 2. Although there are numerical differences in the quantitative results, the trends shown in the middle and right panels reveal the same essence. It can be observed from the middle and right panel that the C–C couples hold the largest fitness during the competition with the help of interdependent networks reciprocity. Note that C–D and D–C couples hold the same average fitness (because $\alpha$ = 0.5 in this article, the payoffs of individuals on two layers of networks contribute the same to the fitness of individuals on one layer of networks). Moreover, all types of couples have maximum fitness at $u = 0.2$. It implies that moderate punishment is not only conducive to the evolution of cooperation, but also to all kinds of couples at the interfaces of cooperators and defectors. The differences in fitness of strategy couples determine their spatial distribution characteristics and the fraction in the system. Specifically, the $F_{C-D}$ curve and the $F_{D-D}$ curve intersect at $u = 0.2$. When $u < 0.2$, the average fitness of C–D couples is higher than D–D couples, and there are more C–D couples in the system. When $u> 0.2$, the average fitness of C–D couples is lower than D–D couples, and there are more D–D couples in the system (it can be observed in Fig. 3).

The color map in Fig. 5 encodes the stationary fraction of cooperators in dependence on the temptation to defect *T* and the punishment factor *u*. Panel (a–d) are obtained with parameter $\alpha =0,0.3,0.6,0.9$, respectively. One can see that, the cooperation rates monotonously increase with the increase of fine, which is different from Fig. 2 when $\alpha =0$. It implies that when two partners are unrelated in payoffs, the presence of peer pressure may still affect the evolution of cooperation, and the punishment effect monotonously conducive to the evolution of cooperation. However, when $\alpha > 0$, the above results are still robust, and there are optimal values of *u* can most effectively promote cooperation. Moreover, it can be observed that the higher the proportion of the partner’s payoff in the individual fitness score, the higher the cooperation rate. It is different from the previous results^32^, which indicate only an intermediate density of sufficiently strong interactions between networks warrants an optimal resolution of social dilemmas. Panel (e) and (f) represent the stationary cooperation rate on the regulate small-world networks and random networks when $\alpha = 0.5$, and the optimal value of *u* can also be observed.

## Discussion

Although peer pressure may have a negative impact on personal life, emotions, etc., it may still have some positive effects on society^24^. Considering that the psychological punishment caused by social pressure may be more affected by a small number of closely related individuals, this work examines the situation of interdependent individuals and analyzes its impact on cooperation. Specifically, partners on interdependent networks will be punished in payoffs when their strategies are inconsistent. Conversely, if the strategy is consistent, a full benefit can be obtained and result in a higher fitness score.

It can be seen from the simulation results that the proportion of interdependent individuals’ inconsistent behavior monotonously decreases with the increase of fines. When $\alpha =0$, individuals on two layers of networks are independent on payoffs, and the punishment effect monotonically promotes cooperation. However, when $\alpha >0$, the monotonic decrease of the inconsistent couples leads to the non-monotonicity promotion on the cooperation rate. The micro result reveals two opposite important influences (protecting cooperators and protecting defectors) of inconsistent couples on the evolution of cooperation. Combined with the other values of $\alpha$, only intermediate fines are most conducive to interdependent network reciprocity. Under the punishment mechanisms, the higher the proportion of the partner’s payoff in the individual fitness score, the higher the cooperation rate. It implies that when partners have a higher degree of interdependence in payoffs, peer pressure can exert a greater effect in enhancing the interdependent network reciprocity. In addition, the small-world and random networks also support the main conclusion in this work.

The punishment mechanism in this paper is to abstract the state of individuals suffering from peer pressure. And through behavior imitation, it spontaneously leads to behavior convergence. This spontaneous behavioral convergence may provide evidence for herd mentality. Moreover, the important conclusion is that only an intermediate fine can maximize cooperation. This shows that although peer pressure is conducive to group collaboration in some cases, it is more necessary to actively guide the situation that is most conducive to society.

The exploration of cooperative behavior proves that the behavioral convergence effect caused by peer pressure is beneficial to network reciprocity and better social cooperation^20^. Finally, we hope this work may give some inspiration to the studies of social pressure and evolutionary cooperation.

## Methods

The prisoner’s dilemma game (*PDG*) participants are arranged on the nodes of two layers of $L \times L$ lattices (with von Neumann neighborhood) with periodic boundaries. Each player has only one coupling partner on another layer of networks. In a standard *PDG*, two players choose to cooperate (*C*) or defect (*D*) simultaneously. They both receive *R* (*P*) for mutual cooperation (defection); if one cooperates while the other defects, the cooperator receives *S*, and the defector receives *T*. $T>R>P>S$ to ensure the essence of the prisoner’s dilemma game, in which the payoff of defection is higher than cooperation, regardless of the opponent’s strategy. Simple but without loss of generality, we set $R = 1$, $T=b$, and $S=P=0$, and $1<b<2$ indicates the defection to defect. In addition, if two interdependent individuals hold different strategies, both of them can only receive $(1-u)$ times of the accumulative payoff. In other words, the parameter *u* represents the degree of punishment of peer pressure on individuals’ payoff.

The simulation is carried out according to the Monte Carlo (*MC*) procedure. The initial state of the evolution is that each participant on the two-layer networks has a random strategy (cooperation or defection). In each basic step of *MC* simulation, players on both layers updates their strategies asynchronously, both players will update their strategy asynchronously, which means that each player has an average chance of imitating strategies from a randomly selected neighbor. The probability that the randomly selected player *x* learns from its randomly selected neighbor player *y* is:

1 $$\begin{aligned} W_{(S_y \rightarrow S_x)} = \frac{1}{1+\exp [-(F_x-F_y)/K]} \end{aligned}$$

where $K = 0.1$ represents the noise factor, $F_x$ and $F_y$ are the fitness of player *x* and player *y*. The fitness of player *x* can be expressed by $F_x = \alpha P_x + (1-\alpha ) P_{x'}$, where $P_x$ and $P_{x'}$ indicate the payoffs of player *x* and his partner $x'$. The parameter $\alpha$ is the coupling coefficient, which indicates the degree of influence that one’s corresponded coupling partner’s payoff to its fitness. For example, when $\alpha = 0$, the individuals on the two-layer networks are independent of each other, and the evolution is essentially on two isolated networks. When $\alpha = 1$, one’s fitness is completely determined by the interaction of his partner on another layer of the network.

In the present paper, all the quantified results are determined with the last 5000 steps of overall 50,000 *MC* steps, and the main quantitative data is averaged for 10 independent simulations.
